# Network Pharmacology-Based and Molecular Docking-Based Analysis of Suanzaoren Decoction for the Treatment of Parkinson's Disease with Sleep Disorder

**DOI:** 10.1155/2021/1752570

**Published:** 2021-10-08

**Authors:** Yan-yun Liu, Li-hua Yu, Juan Zhang, Dao-jun Xie, Xin-xiang Zhang, Jia-ming Yu

**Affiliations:** ^1^Graduate School, Anhui University of Traditional Chinese Medicine, Hefei, Anhui 230038, China; ^2^College of Integrated Chinese and Western Medicine, Anhui University of Traditional Chinese Medicine, Hefei, Anhui 230038, China; ^3^Center of Neurology, Anhui Provincial Hospital of Traditional Chinese Medicine, Hefei, Anhui 230031, China

## Abstract

This study is aimed at exploring the possible mechanism of action of the Suanzaoren decoction (SZRD) in the treatment of Parkinson's disease with sleep disorder (PDSD) based on network pharmacology and molecular docking. Traditional Chinese Medicine Systems Pharmacology (TCMSP) was used to screen the bioactive components and targets of SZRD, and their targets were standardized using the UniProt platform. The disease targets of “Parkinson's disease (PD)” and “Sleep disorder (SD)” were collected by OMIM, GeneCards, and DisGeNET databases. Thereafter, the protein-protein interaction (PPI) network was constructed using the STRING platform and visualized by Cytoscape (3.7.2) software. Then, the DAVID platform was used to analyze the Gene Ontology (GO) enrichment analysis and Kyoto Encyclopedia of Genes and Genomes (KEGG) pathway. Cytoscape (3.7.2) software was also used to construct the network of the “herb-component-target-pathway.” The core active ingredients and core action targets of the drug were verified by molecular docking using AutoDock software. A total of 135 Chinese herbal components and 41 corresponding targets were predicted for the treatment of PDSD using SZRD. Fifteen important signaling pathways were screened, such as the cancer pathway, TNF signaling pathway, PI3K-AKT signaling pathway, HIF-1 signaling pathway, and Toll-like receptor signaling pathway. The results of molecular docking showed that the main active compounds could bind to the representative targets and exhibit good affinity. This study revealed that SZRD has the characteristics and advantages of “multicomponent, multitarget, and multipathway” in the treatment of PDSD; among these, the combination of the main active components of quercetin and kaempferol with the key targets of *AKT1*, *IL6*, *MAPK1*, *TP53*, and *VEGFA* may be one of the important mechanisms. This study provides a theoretical basis for further study of the material basis and molecular mechanism of SZRD in the treatment of PDSD.

## 1. Introduction

Parkinson's disease (PD) is a common neurodegenerative disease in middle-aged and elderly individuals. Clinically, symptoms include classic motor symptoms—tremor, myotonia, bradykinesia, and postural imbalance—as well as nonmotor symptoms—sleep disorders, smell disorders, autonomic nervous dysfunction, and cognitive and mental disorders [1]. Sleep disorders (SD), the most common nonmotor symptoms of PD, seen in 60–90% of these patients, are one of the common nocturnal symptoms [2]. Treatment modalities of PD with sleep disorder (PDSD), used in clinical practice, include levodopa, dopamine receptor agonists, benzodiazepines, and melatonin [3–5]. However, long-term use of such drugs may enhance restless leg syndrome, periodic limb movements, and rapid eye movement (REM) sleep behavior disorder symptoms [6]. Furthermore, melatonin had little effect on objective sleep parameters [7]. Suanzaoren decoction (SZRD), derived from JinKuiYaoLue, is composed of five traditional Chinese medicines: *Semen* ziziphi spinosae, *Glycyrrhiza glabra*, *Rhizoma anemarrhenae*, *Poria cocos*, and *Rhizoma chuanxiong* [8]. It has the effect of nourishing the blood, calming the mind, clearing away heat, and eliminating annoyance, mainly treating liver and blood deficiency and insomnia caused by heat deficiency [9–12]. PD belongs to the category of “fibrillation disease” in traditional Chinese medicine, and it is more common in the elderly [13]. Clinical evidence shows that SZRD has a remarkable curative effect on insomnia [14–16]. However, the mechanism of action of fibrillation disease merging with wakefulness is not clear. Therefore, with the systematic research methods of network pharmacology and molecular docking, the overall analysis of the “herb-component-target-pathway” was conducted in this study to explore the possible mechanism of SZRD in the treatment of PDSD and to provide new theoretical support for the clinical treatment of PDSD.

## 2. Materials and Methods

### 2.1. Screening and Target Prediction of Active Components of SZRD

Application analysis platform and database system pharmacology of Chinese medicine (TCMSP, https://tcmspw.com/tcmsp.php) [17] were used to retrieve active ingredients of SZRD and predict the targets of active ingredients. We use pharmacodynamics to select active ingredients satisfying both oral bioavailability (OB) ≥ 30% and drug − likeness (DL) ≥ 0.18 [18, 19]. TCMSP was used for the prediction of targets of active ingredients. At the same time, the UniProt database (https://www.uniprot.org/) [20], which was set for human species, was used to standardize the drug target of each active ingredient.

### 2.2. Screening of Disease-Related Targets

The targets related to the PD and SD were obtained through retrieving the OMIM (https://omim.org/search/advanced/) [21], GeneCards (https://www.genecards.org/) [22], and DisGeNET database search (https://www.disgenet.org/) [23] using the keyword “Parkinson's diseases” or “Sleep disorder.”

### 2.3. Screening of Common Targets of Diseases and Drugs

The online Venny 2.1 mapping platform (http://bioinfogp.cnb.csic.es/tools/venny/index.html) was used to map “Parkinson's diseases,” “Sleep disorder,” and “SZRD” targets and to get the intersection targets.

### 2.4. Common Target PPI Network Construction

The intersection targets were imported into the STRING database (https://string-db.org/cgi/input) [24]. A confidence, ≥0.4, was taken, and the free nodes were hidden to construct the protein-protein interaction (PPI) network. This PPI network was further processed by Cytoscape 3.7.2 software [25] to realize visualization and screen out the core targets.

### 2.5. GO and KEGG Enrichment Analysis

For the screened core targets, the DAVID data platform (https://david.ncifcrf.gov/tools.jsp) [26] was used for Gene Ontology (GO) functional annotations and Kyoto Encyclopedia of Genes and Genomes (KEGG) pathway enrichment analysis. Select “Homo species” on the DAVID platform; further analyze the SZRD for PDSD-related biological processes (BP), cellular component (CC), molecular function (MF), and signal pathway; and use the bioinformatic online platform (https://www.bioinformatics.com.cn/) [27] to visualize the result analysis.

### 2.6. Constructing the “H-C-T-P” Network

Cytoscape 3.7.2 software was used to construct the network of “herb-component-target-pathway” (H-C-T-P). This “H-C-T-P” network together with the screened main active ingredients, core targets, and concentrated main signal pathways was used to systematically analyze the possible mechanism of SZRD in the treatment of PDSD.

### 2.7. Docking and Verification of Potential Active Ingredients with Core Target Molecules

The 2D structures of the potential active ingredients in SZRD were downloaded from the TCMSP database while the 3D structure of the PDSD docking targets (top 5 of degree in PPI network) treated by SZRD was downloaded from the Worldwide Protein Data Bank (PDB) database (https://www.rcsb.org/) [28]. They were imported to AutoDockTools [29] for hydrogenation, dehydration, and other pretreatments. Then, molecular docking of the receptor and ligand was conducted to analyze its binding activity. The docking results were visualized using the PyMol software [30].

## 3. Results

### 3.1. The Active Components and Effective Targets of SZRD

By searching the TCMSP database, 135 different active ingredients of SZRD were screened, including 9 in *Semen* ziziphi spinosae, 15 in *Poria cocos*, 15 in *Rhizoma anemarrhenae*, 7 in *Rhizoma chuanxiong*, and 92 in *Glycyrrhiza glabra*. The results showed that these different drugs contained common active ingredients, namely A, B, and C as shown in Table 1.

Cytoscape 3.7.2 software was used to screen the active ingredients with a degree ≥ 20 in SZRD. As shown in Table 2, 17 major components, which include quercetin, kaempferol, vestitol, 7-methoxy-2-methyl isoflavone, naringenin, anhydroicaritin, formononetin, stigmasterol, licochalcone A, and isorhamnetin, were obtained using this software.

**Figure 1:**
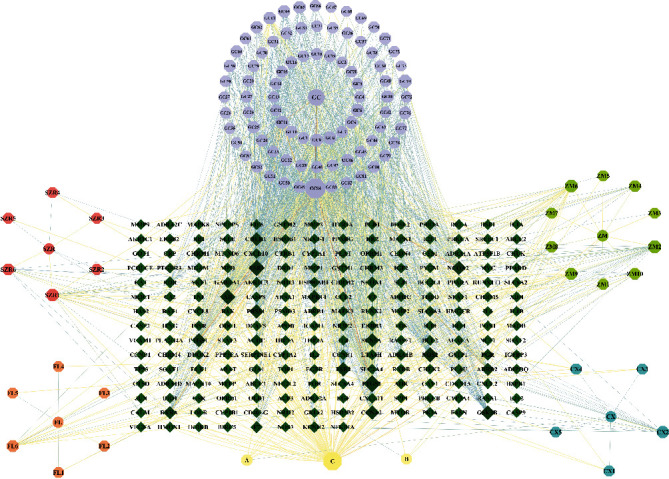
Composition-target network of SZRD. Circles are for traditional Chinese medicine; octagons are compound component; diamonds are target. GC: *Glycyrrhiza glabra*; SZR: *Semen* ziziphi spinosae; FL: *Poria cocos*; ZM: *Rhizoma anemarrhenae*; CX: *Rhizoma chuanxiong*; A: common components of *Glycyrrhiza glabra* and *Rhizoma chuanxiong*; B: common components of *Glycyrrhiza glabra* and *Semen* ziziphi spinosae; C: common components of *Glycyrrhiza glabra* and *Rhizoma anemarrhenae*.

A total of 204 different drug targets were screened using the TCMSP database and were standardized by the UniProt database, which included the 26 in *Semen* ziziphi spinosae, 16 in *Poria cocos*, 90 in *Rhizoma anemarrhenae*, 23 in *Rhizoma chuanxiong*, and 193 in *Glycyrrhiza glabra*. The data of potential active ingredients and potential targets of SZRD in the treatment of PDSD were imported into Cytoscape 3.7.2 software to obtain a diagram of the traditional Chinese medicine component-target network (Figure 1).

### 3.2. Related Targets for Disease

After combining the three databases and deleting repeated targets, a total of 9777 PD-related targets and 10748 SD-related targets were obtained from the OMIM, DisGeNET, and GeneCards databases.

### 3.3. Common Targets for Diseases and Drugs

**Figure 2:**
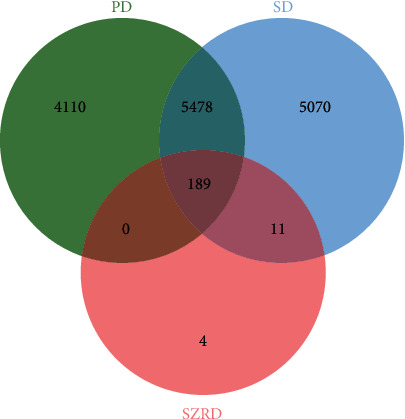
Disease-drug target Venn diagram.

A total of 9777 PD-related targets, 10748 SD-related targets, and 204 SZRD drug prediction targets were imported using the Venny online mapping platform. After mapping, 189 intersection targets of SZRD and PDSD were obtained (Figure 2).

### 3.4. PPI Network Construction

**Figure 3:**
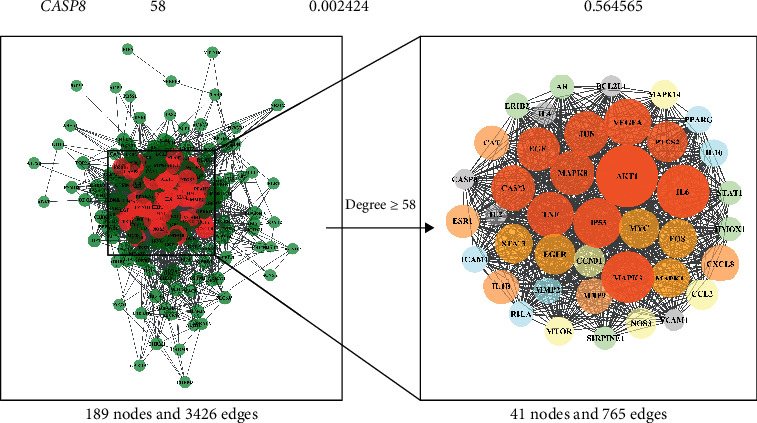
Core target PPI network. As the figure shows, the larger area of the circle could be considered as more important in this network.

A total of 189 targets were imported into the STRING platform to construct a PPI network. Then, 189 nodes and 3426 edges were also obtained using this platform. The double median of “Degree,” that is, “Degree ≥58,” was used to screen the intersection targets. Thus, 41 nodes, 765 edges, and a total of 41 core targets for SZRD treatment of PDSD were obtained (Table 3). Import the PPI network information obtained from the STRING platform into Cytoscape (3.7.2) software for visualization (Figure 3).

### 3.5. GO and KEGG Enrichment Analysis

**Figure 4:**
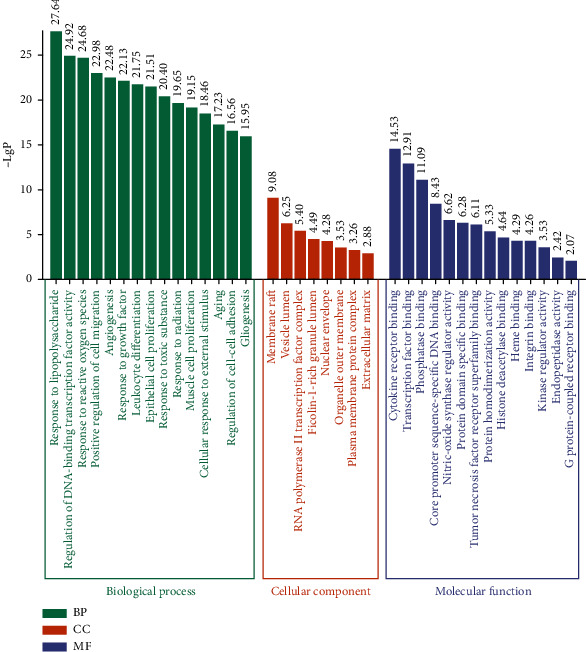
GO function enrichment results of SZRD in the treatment of PDSD.

The GO function enrichment analysis of the 41 core targets was performed on the DAVID platform, and a total of 1423 GO items were obtained, including 1325 BP, 36 CC, and 62 MF. The first 15, 8, and 14 items were selected based on the *P* value for visual analysis (Figure 4). Results showed that the treatment of PDSD by SZRD mainly involves BP such as cell migration, angiogenesis, leukocyte differentiation, cell proliferation, stress, cell aging, cell adhesion, and cell regeneration. These targets pass through cytokine receptor binding, transcription factor binding specificity, phosphatase binding, DNA binding domain specificity, protein structure combining, G protein coupled receptor molecules, and other functions, and they play a role in the cell membrane, RNA polymerase II transcription factor complex, nucleus and organelle outer membrane and plasma membrane protein complexes, and extracellular matrix components.

**Figure 5:**
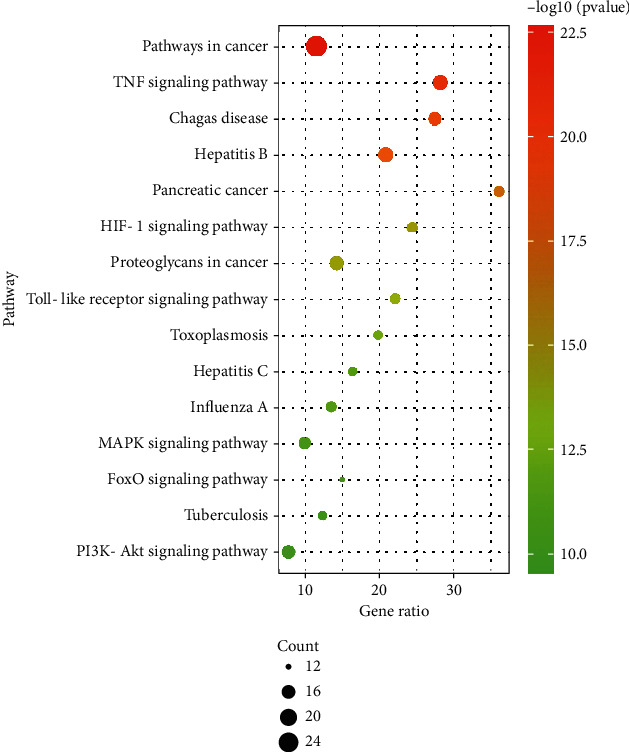
KEGG enrichment bubble diagram.

One hundred and nine signal pathways were enriched by KEGG pathway analysis of the core targets using the DAVID platform. According to the *P* value < 0.05 and the number of genes ≥ 12, 15 signal pathways with high probability were screened out for visual analysis as shown in Table 4 and Figure 5. Moreover, Figure 5 shows that SZRD treatment of PDSD may be mainly related to TNF, PI3K-Akt, MAPK, HIF-1, Toll-like receptor, FoxO, and other signaling pathways.

### 3.6. Construction of “H-C-T-P” Network

**Figure 6:**
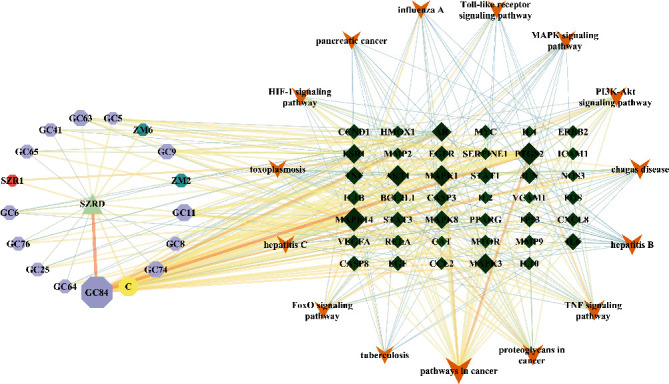
H-C-T-P network diagram. Triangle is SZRD; octagons are compound component; diamonds are target; V is pathway.

The “H-C-T-P” network was constructed using the 17 major components, 41 core targets, and 15 signal pathways of SZRD in the treatment of PDSD (Figure 6).

### 3.7. Molecular Docking Results and Analysis

**Figure 7:**
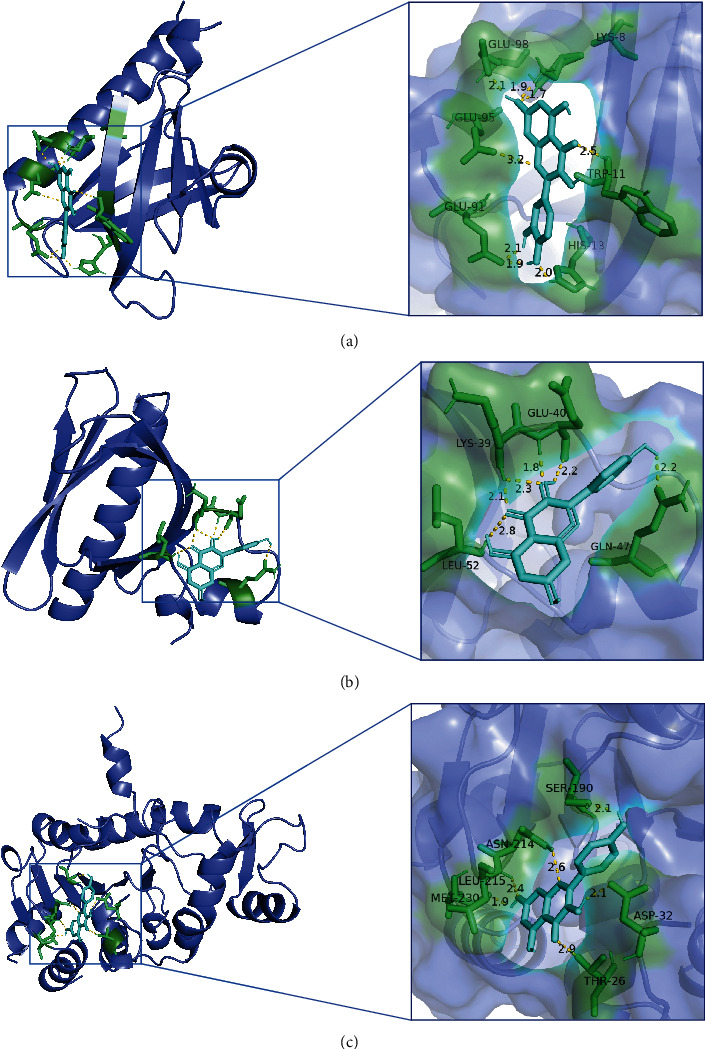
Molecular docking diagram of chemical composition to target: (a) 1UNQ-quercetin; (b) 1UNQ-kaempferol; (c) 1YC5-kaempferol.

According to Table 3, the top 5 targets of degree are AKT1, IL6, MAPK1, TP53, and VEGFA. The docking targets with the two active components with the highest degree of quercetin (degree = 127) and kaempferol (degree = 104) in SZRD were docking. As shown in Table 5, the binding energy of quercetin, kaempferol with AKT1, IL6, MAPK1, TP53, and VEGFA was all less than -5.0 kcal·mol^−1^, showing good binding force. The binding of AKT1 to quercetin and kaempferol and TP53 to kaempferol is shown in Figure 7.

## 4. Discussion

### 4.1. Understanding of PDSD in Traditional Chinese Medicine and Western Medicine

In Western medicine, PDSD is believed to be associated with a variety of factors which may also be related to the increase or decrease of serum vitamin D, melatonin, serum cystatin (Cys) C, homocysteine (Hcy), and dopamine levels in the striatum caused by PD itself [31–34].

There is no related record of PDSD in traditional Chinese medicine classes, but according to its related clinical symptoms, it can be classified as a combination of “fibrillation” and “insomnia.” SZRD is a classic prescription, with tranquilizing properties, mainly treating “deficiency of liver and blood, deficiency of heat, and internal disturbance.” This prescription uses a lot of *Semen* ziziphi spinosae that nourishes the blood and liver and gives peace of mind. *Rhizoma anemarrhenae* nourishes Yin and clears the heat, and when the evil heat is gone, the healthy Qi is restored. “*Poria cocos* calms the heart and tranquilizes the mind.” Moreover, the theory of the properties of drugs says it is “good at calming the mind.” *Rhizoma anemarrhenae* and *Poria cocos* are used to help *Semen* ziziphi spinosae calm the mind from vexation. The nature of *Rhizoma chuanxiong* is scattered, “Qi medicine in the blood,” and is introduced into the liver meridian. Furthermore, this regulates Qi activity to help the liver recover its ability to dredge and release Qi. Rihua Zi medicine said that it “cures all wind, all Qi, all strain, all blood, and replenish five kinds of weakness.” *Rhizoma chuanxiong* is used in combination with King medicine, which has a magical effect to help sanguification and liver recuperation. Then, *Glycyrrhiza glabra* is used to harmonize the various drugs. All the drugs work together, to calm the mind in addition to the effect of vexation.

### 4.2. The Mechanism Prediction of SZRD for PDSD

Based on the network pharmacological research method, a total of 17 major active components of SZRD, which include quercetin, kaempferol, visistin, 7-methoxy-2-methyl isoflavone, naringenin, anhydroicaritin, formononetin, stigmasterol, licochalcone A, and isorhamnetin, in the treatment of PDSD were obtained.

Quercetin, kaempferol, and isorhamnetin are flavonoids which can scavenge reactive oxygen free radicals and achieve antioxidant and neuroprotective effects [35–38]. In the H_2_O_2_-induced PD model PC-12 cells, quercetin treatment increased cell viability, reduced mutagenesis of antioxidant enzymes, and reduced apoptosis of cells and hippocampal neurons [39]. In addition, quercetin can significantly reduce inflammation and oxidative damage in the striatum of PD model rats induced by 6-hydrox-ydopamine (6-OHDA) or 1-methyl-4-phenyl-1,2,3,6-tetrahydropyridine (MPTP) [40]. Filomeni et al. found that kaempferol can significantly protect neuroblastoma cells (SH-SY5Y nerve cells) and major neurons from rotenone damage [41]. Moreover, it can reduce protease lysis and nuclear apoptosis and significantly reduce oxidative stress levels and mitochondrial hydroxyl compound content [41]. We also found that kaempferol may play a neuroprotective role by inhibiting cysteine proteinase-3 and by reducing brain cell apoptosis [42]. At the same time, quercetin and kaempferol have sedative and hypnotic effects, and both can also improve the effects of sleep disorders [43–46].

According to the GO and KEGG pathway enrichment analysis results, we speculated that the mechanism of SZRD in the treatment of PDSD may be mainly related to TNF, PI3K-Akt, MAPK, HIF-1, Toll-like receptors, and FoxO-associated signaling pathways. Such as involving *AKT1*, *IL6*, *MAPK3*, *TP53*, and *VEGFA* multiple key targets, cell aging, adhesion, regeneration, and angiogenesis, leukocyte differentiation is closely related to the process of cell metabolism. Recent studies have shown that PI3K-Akt, MAPK, and FOXO are all involved in the process of human cell apoptosis [47–49]. AKT1, an important member of the AKT (protein kinase B, PKB) family, is an intracellular serine/threonine involved in a variety of cellular BP, and its activation is mainly dependent on the PI3K signaling pathway [50]. AKT1 is activated by phosphorylation, which in turn activates downstream signaling molecules [51]. The PI3K/Akt signal transduction pathway is involved in a variety of cytokines, and it has been found that by reducing the phosphorylation levels of PI3K, AKT, and mTOR, the transduction of this pathway can play a role in the treatment of MPTP-induced PD mice [52]. Studies have shown that cytokines are involved in regulating sleep, among which interleukin-1 (IL-1), interleukin-6 (IL-6), and tumor necrosis factor (TNF-*α* and TNF-*β*) play a role in promoting sleep [53–55]. Elevation of interleukin-1*β* (IL-1*β*) and TNF-*α* can directly act on central sleep-wake neurons, causing increased nonrapid eye movement (NREM) sleep and regulating the pathologic circadian rhythm [56, 57]. IL-1*β* and TNF-*α*, as sleep-regulating cytokines, can induce the production of each other [58]. After the activation of the PI3K-Akt signaling pathway, the activity of inflammatory mediator genes was upregulated, which promoted the production of a large number of cytokines and the increase in the levels of TNF-*α*, IL-6, and IL-1*β*, thus playing a role in regulating sleep [59]. Mitogen-activated protein kinase (MAPK) is a kind of serine/threonine protein kinases in cells. The MAPK-mediated signal transduction pathway is mainly related to the inflammatory response and plays a role in the phosphorylation of protein c-Jun [60]. Studies have shown that SP600125, a specific inhibitor of the JNK signaling pathway, significantly reduced the expression of phosphorylated c-Jun in the substantia nigra of the midbrain in PD model mice [61]. It was found that MAPK, AKT1, and IL-6 in the serum of patients with anxiety were all increased, and there was a significant positive correlation between anxiety severity and sleep quality. With the improvement of anxiety state and sleep state, the expression of MAPK decreases gradually [62–64]. The FOXO signaling pathway is interrelated with the PI3K-Akt and MAPK signaling pathways [65, 66]. FoxO3a protein is an important subtype of the FoxO family and is an important cytokine in PI3K-Akt signal transduction [67]. After phosphorylation and modification of FoxO3a, specific downstream target genes can be activated to induce autophagy and apoptosis of cells [68]. For example, when the MAPK pathway is activated or the PI3K-Akt pathway is inhibited, FoxO3a is dephosphorylated, thereby regulating different downstream factors and promoting cell apoptosis [68]. HIF-1 and its target gene *VEGF* can play a protective role in PD through mechanisms such as antioxidant stress, and the overexpression of VEGF can promote the proliferation and differentiation of neurons and reduce MPTP-induced substantia nigra cell injury [69]. The expression of angiogenic factor VEGF increased with an increase in the severity of sleep disturbance [70].

## 5. Conclusions

In this study, we used the network pharmacology research method to predict the chemical composition, target, and signal pathways at multiple levels. The prediction results were verified by molecular docking technology. The results show that SZRD plays an important role in the treatment of sleep disorders associated with PD through “multicomponent, multitarget, and multipathway,” which provides a new theoretical basis for further experimental research and clinical treatment.

## Figures and Tables

**Table 1:** SZRD shares chemical composition information table.

Number	Mol ID	Molecule name	OB%	DL	Drug
A	MOL000359	Sitosterol	36.91	0.75	*Glycyrrhiza glabra*, *Rhizoma chuanxiong*
B	MOL000211	Mairin	55.38	0.78	*Glycyrrhiza glabra*, *Semen* ziziphi spinosae
C	MOL000422	Kaempferol	41.88	0.24	*Glycyrrhiza glabra*, *Rhizoma anemarrhenae*

**Table 2:** Chemical information sheet of major active ingredients.

Number	Mol ID	Molecule name	OB%	DL	Degree
GC84	MOL000098	Quercetin	46.43	0.28	127
C	MOL000422	Kaempferol	41.88	0.24	104
GC74	MOL000500	Vestitol	74.66	0.21	33
GC8	MOL003896	7-Methoxy-2-methyl isoflavone	42.56	0.20	30
GC11	MOL004328	Naringenin	58.29	0.21	29
ZM2	MOL004373	Anhydroicaritin	45.41	0.44	27
GC9	MOL000392	Formononetin	69.67	0.21	26
ZM6	MOL000449	Stigmasterol	43.48	0.76	24
GC5	MOL002565	Medicarpin	49.22	0.34	24
GC63	MOL000497	Licochalcone a	40.79	0.29	24
GC41	MOL004891	Shinpterocarpin	80.3	0.73	23
GC65	MOL004978	2-[(3R)-8,8-Dimethyl-3,4-dihydro-2H-pyrano[6,5-f] chromen-3-yl]-5-methoxyphenol	36.21	0.52	23
SZR1	MOL001522	(S)-Coclaurine	42.35	0.24	22
GC6	MOL004980	Isorhamnetin	39.71	0.33	22
GC76	MOL005003	Licoagrocarpin	58.81	0.58	21
GC25	MOL004835	Glypallichalcone	61.60	0.19	20
GC64	MOL004974	3′-Methoxyglabridin	46.16	0.57	20

GC: *Glycyrrhiza glabra*; C: common components of *Glycyrrhiza glabra* and *Rhizoma anemarrhenae*; ZM: *Rhizoma anemarrhenae*; SZR: *Semen* ziziphi spinosae.

**Table 3:** Core target information table.

Target	Degree	Betweenness centrality (BC)	Closeness centrality (CC)
AKT1	123	0.064225	0.731518
IL6	110	0.033377	0.693727
MAPK3	109	0.052416	0.693727
TP53	105	0.027917	0.676259
VEGFA	100	0.016309	0.664311
TNF	98	0.018693	0.661972
CASP3	97	0.019316	0.661972
JUN	97	0.021842	0.664311
EGF	95	0.020902	0.657343
MAPK8	95	0.017033	0.657343
PTGS2	91	0.032141	0.646048
MAPK1	90	0.012068	0.639456
EGFR	89	0.016470	0.641638
MYC	89	0.013782	0.639456
STAT3	88	0.011530	0.635135
FOS	87	0.033525	0.639456
CXCL8	85	0.016357	0.626667
MMP9	85	0.027723	0.632997
IL1B	82	0.013500	0.626667
CAT	81	0.038221	0.624585
ESR1	79	0.010994	0.616393
CCND1	77	0.006779	0.610390
CCL2	76	0.005212	0.608414
NOS3	74	0.020902	0.614379
MTOR	71	0.006984	0.598726
MAPK14	71	0.005773	0.600639
IL10	70	0.003254	0.593060
MMP2	69	0.003821	0.596825
PPARG	68	0.008861	0.594937
ICAM1	67	0.002937	0.591195
RELA	67	0.014775	0.585670
ERBB2	66	0.009451	0.587500
AR	64	0.017042	0.589342
HMOX1	63	0.006109	0.580247
STAT1	63	0.006329	0.574924
SERPINE1	62	0.004200	0.580247
IL4	61	0.002350	0.576687
IL2	60	0.002819	0.569697
VCAM1	60	0.002099	0.574924
BCL2L1	59	0.002173	0.567976
CASP8	58	0.002424	0.564565

**Table 4:** KEGG pathway enrichment results.

Term	%	Count	*P* value	Related genes
hsa05200: pathways in cancer	65.8536	27	2.30*E*-23	*CXCL8*, *PTGS2*, *RELA*, *EGFR*, *MAPK8*, *CASP8*, *CCND1*, *MYC*, *CASP3*, *ERBB2*, *AKT1*, *MAPK1*, *MAPK3*, *JUN*, *EGF*, *STAT1*, *MMP2*, *STAT3*, *FOS*, *MMP9*, *MTOR*, *VEGFA*, *AR*, *IL6*, *PPARG*, *TP53*, *BCL2L1*
hsa04668: TNF signaling pathway	43.9024	18	3.19*E*-21	*JUN*, *VCAM1*, *FOS*, *PTGS2*, *MAPK14*, *TNF*, *MMP9*, *RELA*, *ICAM1*, *IL6*, *MAPK8*, *CASP8*, *IL1B*, *CASP3*, *CCL2*, *AKT1*, *MAPK1*, *MAPK3*
hsa05161: hepatitis B	43.9024	18	7.08*E*-19	*JUN*, *CXCL8*, *STAT1*, *STAT3*, *FOS*, *TNF*, *MMP9*, *RELA*, *IL6*, *MAPK8*, *CASP8*, *CCND1*, *MYC*, *CASP3*, *AKT1*, *MAPK1*, *TP53*, *MAPK3*
hsa05142: Chagas disease	41.4634	17	1.04*E*-19	*IL10*, *JUN*, *CXCL8*, *SERPINE1*, *FOS*, *MAPK14*, *TNF*, *IL2*, *RELA*, *IL6*, *MAPK8*, *CASP8*, *IL1B*, *CCL2*, *AKT1*, *MAPK1*, *MAPK3*
hsa05205: proteoglycans in cancer	41.4634	17	4.91*E*-15	*MMP2*, *STAT3*, *MAPK14*, *ESR1*, *TNF*, *MMP9*, *EGFR*, *MTOR*, *VEGFA*, *CCND1*, *MYC*, *CASP3*, *ERBB2*, *AKT1*, *MAPK1*, *TP53*, *MAPK3*
hsa04151: PI3K-Akt signaling pathway	39.0243	16	3.03*E*-10	*NOS3*, *EGF*, *EGFR*, *IL2*, *MTOR*, *RELA*, *VEGFA*, *IL4*, *IL6*, *CCND1*, *MYC*, *AKT1*, *MAPK1*, *TP53*, *BCL2L1*, *MAPK3*
hsa04010: MAPK signaling pathway	36.5853	15	5.72*E*-11	*JUN*, *EGF*, *FOS*, *MAPK14*, *TNF*, *EGFR*, *RELA*, *MAPK8*, *MYC*, *IL1B*, *CASP3*, *AKT1*, *MAPK1*, *TP53*, *MAPK3*
hsa05212: pancreatic cancer	34.1463	14	1.31*E*-17	*STAT1*, *EGF*, *STAT3*, *EGFR*, *RELA*, *VEGFA*, *MAPK8*, *CCND1*, *ERBB2*, *AKT1*, *MAPK1*, *TP53*, *BCL2L1*, *MAPK3*
hsa04066: HIF-1 signaling pathway	34.1463	14	2.96*E*-15	*NOS3*, *EGF*, *STAT3*, *SERPINE1*, *EGFR*, *MTOR*, *RELA*, *VEGFA*, *IL6*, *ERBB2*, *AKT1*, *MAPK1*, *HMOX1*, *MAPK3*
hsa04620: Toll-like receptor signaling pathway	34.1463	14	1.11*E*-14	*JUN*, *CXCL8*, *STAT1*, *FOS*, *MAPK14*, *TNF*, *RELA*, *IL6*, *MAPK8*, *CASP8*, *IL1B*, *AKT1*, *MAPK1*, *MAPK3*
hsa05164: influenza A	34.1463	14	7.36*E-*12	*JUN*, *CXCL8*, *STAT1*, *MAPK14*, *TNF*, *RELA*, *ICAM1*, *IL6*, *MAPK8*, *IL1B*, *CCL2*, *AKT1*, *MAPK1*, *MAPK3*
hsa05145: toxoplasmosis	31.7073	13	5.84*E*-13	*IL10*, *STAT1*, *STAT3*, *MAPK14*, *TNF*, *RELA*, *MAPK8*, *CASP8*, *CASP3*, *AKT1*, *MAPK1*, *BCL2L1*, *MAPK3*
hsa05160: hepatitis C	31.7073	13	5.83*E*−12	*CXCL8*, *STAT1*, *EGF*, *STAT3*, *MAPK14*, *TNF*, *EGFR*, *RELA*, *MAPK8*, *AKT1*, *MAPK1*, *TP53*, *MAPK3*
hsa05152: tuberculosis	31.7073	13	1.73*E*-10	*IL10*, *STAT1*, *MAPK14*, *TNF*, *RELA*, *IL6*, *MAPK8*, *CASP8*, *IL1B*, *CASP3*, *AKT1*, *MAPK1*, *MAPK3*
hsa04068: FoxO signaling pathway	29.2682	12	1.45*E*-10	*IL10*, *IL6*, *MAPK8*, *CCND1*, *EGF*, *STAT3*, *CAT*, *MAPK1*, *AKT1*, *MAPK14*, *EGFR*, *MAPK3*

**Table 5:** Docking results of target protein and active compound.

Core target	PDB ID	Binding energy/(kcal Mol-1)	
		Quercetin	Kaempferol
AKT1	1UNQ	-6.18	-6.51
IL-6	1ALU	-5.72	-5.90
MAPK3	4QTB	-5.45	-5.33
TP53	1YC5	-5.92	-6.08
VEGFA	4QAF	-5.61	-5.67

## Data Availability

The data used to support the findings of this study are available from the corresponding author upon request.
